# Effective division of the intersegmental plane using a robotic stapler in robotic pulmonary segmentectomy

**DOI:** 10.1007/s00595-024-02840-y

**Published:** 2024-04-18

**Authors:** Mikio Okazaki, Ken Suzawa, Kazuhiko Shien, Kohei Hashimoto, Shin Tanaka, Kentaroh Miyoshi, Hiromasa Yamamoto, Seiichiro Sugimoto, Shinichi Toyooka

**Affiliations:** Department of General Thoracic Surgery and Breast and Endocrinological Surgery, Graduate School of Medicine, Dentistry and Pharmaceutical Sciences, Okayama University, 2-5-1 Shikata-cho, Kita-ku, Okayama 700-8558 Japan

**Keywords:** Pulmonary segmentectomy, Robot-assisted thoracic surgery, Robotic segmentectomy, Robotic stapler

## Abstract

**Purposes:**

Robot-assisted thoracoscopic (RATS) segmentectomy is becoming increasingly common because of the expanded indications for segmentectomy and the widespread adoption of robotic surgery. The precise division of the intersegmental plane is necessary to ensure oncologic margins from the tumor and to preserve the lung function. In this study, we present a strategy for accurately dividing the intersegmental plane using a robotic stapler and review the surgical outcomes.

**Methods:**

RATS portal segmentectomy was performed using the Da Vinci Xi system and the intersegmental plane was dissected using a robotic stapler. We evaluated the perioperative outcomes in 92 patients who underwent RATS portal segmentectomy between May 2020 and January 2023. These results were compared with those of 82 patients who underwent complete video-assisted thoracoscopic surgery (CVATS) during the same period.

**Results:**

The operative and console times were 162 and 97 min, respectively. No intraoperative complications occurred, and postoperative complications were observed in four cases (4.3%). The operative time, blood loss, postoperative complications, and maximum incision size were significantly lower in the RATS group than in the CVATS group. However, RATS requires a significantly higher number of staplers than CVATS.

**Conclusions:**

The division of the intersegmental plane using a robotic stapler in RATS portal segmentectomy was, therefore, found to be safe and effective.

**Supplementary Information:**

The online version contains supplementary material available at 10.1007/s00595-024-02840-y.

## Introduction

Pulmonary segmentectomy is becoming increasingly common because of the expanded indications for early stage-lung cancer. Saji et al. showed that the overall survival of patients who underwent segmentectomy was significantly better than that of patients who underwent lobectomy for non-small cell lung cancer (NSCLC) smaller than 2 cm [1]. However, the study also found a higher incidence of local recurrence in patients who underwent segmentectomy than in those who underwent lobectomy. One potential reason for this local recurrence may be the recurrence of the surgical stump at the intersegmental plane. The precise division of the intersegmental plane is crucial to ensure oncological margins from the tumor and preserve the remaining lung function.

Minimally invasive approaches have become the standard treatment for early-stage lung cancer. Robotic surgery is also widely used in pulmonary and pulmonary segmentectomies [2]. However, in robotic surgery, it is difficult to confirm the surgical margin with the fingers, and greater skill is required for precise division of the intersegmental plane compared to the thoracotomy approach.

While many studies have compared robotic staplers to endoscopic linear staplers in gastric and colorectal robotic surgery, only one study has examined the utility of robotic staplers in pulmonary robotic surgery [3–6]. Zervos et al. compared robot-assisted thoracoscopic (RATS) lobectomy cases with robotic staplers to those with hand-held staplers and reported significantly lower intraoperative bleeding, conversion, postoperative air leaks, and overall complications in robotic stapler cases [3]. However, most staplers are used to dissect pulmonary vessels and bronchi during pulmonary lobectomy. Furthermore, the usefulness of robotic staplers during robotic segmentectomy has not yet been reported. We herein describe the usefulness of a robotic stapler during RATS segmentectomy and strategies for the precise division of the intersegmental plane by leveraging the features of the robotic stapler.

## Patients and methods

### Ethics statement

This retrospective study was approved by the Ethics Committee of Okayama University Graduate School of Medicine, Dentistry and Pharmaceutical Sciences, Okayama University, Okayama, Japan (approval date: 17 March 2023, approval number: 2304–014), and written informed consent was waived.

### Patients

Ninety-two patients who underwent robotic pulmonary segmentectomies and eighty-two patients who underwent complete video-assisted thoracoscopic surgery (CVATS) at Okayama University Hospital between April 2020 and January 2023 were enrolled in this study. The data collected from the patients’ medical records included age, sex, surgery, histology, and treatment course. Postoperative complications were evaluated using the Clavien–Dindo classification system. “Grade II or more” was defined as postoperative complications. A prolonged air leak was defined as an air leak lasting longer than 7 days or when pleurodesis was performed.

### RATS portal segmentectomy

**Fig. 1:**
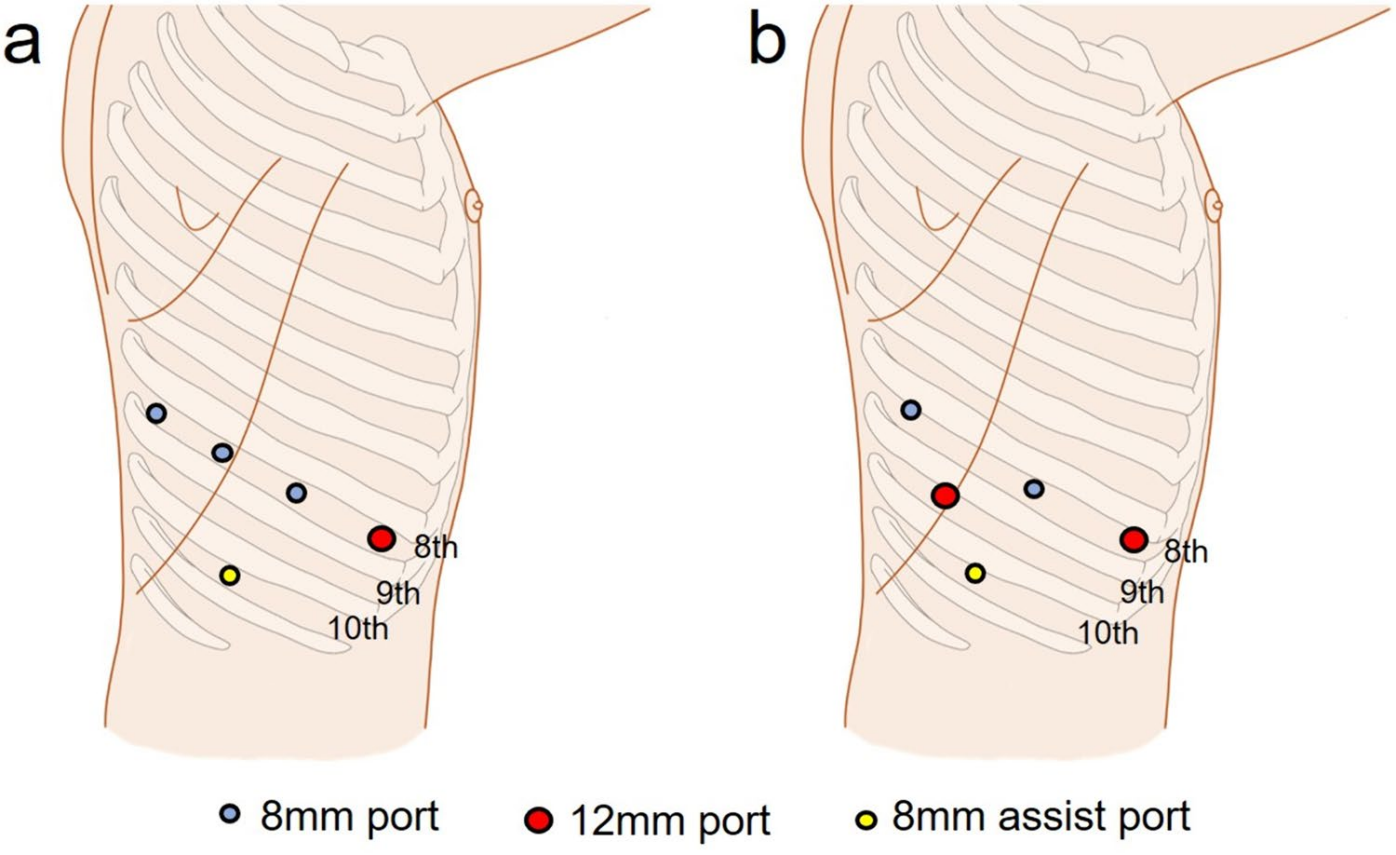
The images of port placement. **a** SureForm can be used via a 12 mm robotic port placed on the ventral side. **b** SureForm can be used from two ports. The second port at the back is 12 mm and is placed on the ninth intercostal space

**Fig. 2:**
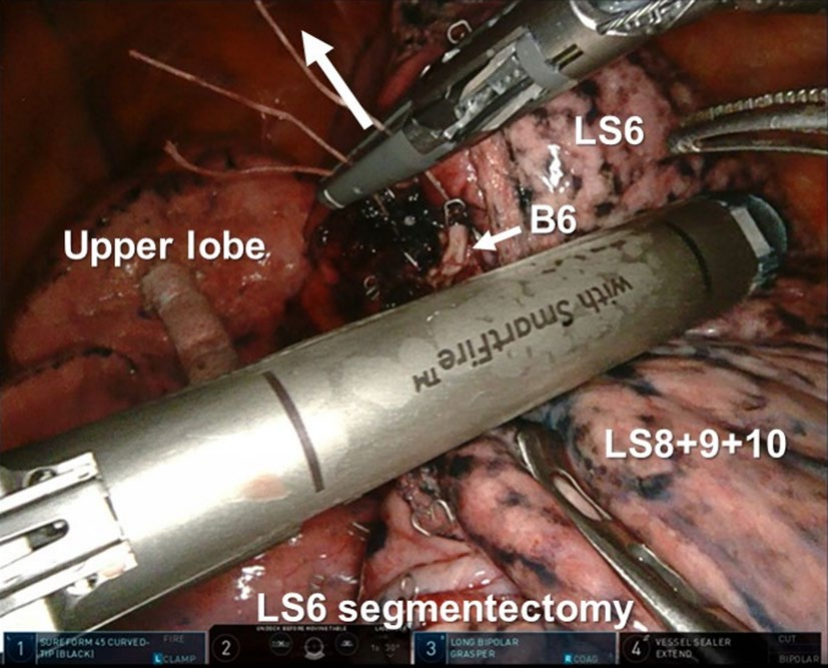
RATS left S6 segmentectomy was performed. During dissection of the pulmonary hilum, the 1–0 silk threads stapled with the blood vessels and bronchi were pulled to outline the correct line of dissection

RATS portal segmentectomies with four robotic arms were performed using the da Vinci Xi surgical system (Intuitive Surgical, Sunnyvale, CA, USA) in the present study. The five robotic instruments used included a 30° camera, Long Bipolar Grasper, Cadiere Forceps, Vessel Sealer and SureForm Stapler 45. Robotic ports were placed in the 8th intercostal space. The 8 mm AirSeal (ConMed, Utica, NY, USA) was placed on the 10th intercostal space, and carbon dioxide (CO_2_) insufflation was performed at 5–8 mmHg through this port. The SureForm was used via a 12 mm robotic port placed on the ventral side (Fig. 1a). In cases involving complex segmentectomies where SureForm was used from two ports, the second port at the back was 12 mm and was placed on the ninth intercostal space (Fig. 1b). Pulmonary vessels and bronchi were exposed, taped with 1–0 silk threads, and divided using a SureForm Stapler 45. 1–0 silk threads were intentionally stapled with these structures using a stapler. To identify the intersegmental line, indocyanine green (ICG) (15 mg) was injected through the peripheral venous catheter, followed by a 10 mL flush of sterile normal saline. The integrated fluorescence imaging capability of the Firefly Fluorescence Imaging camera (Intuitive Surgical) was used to visualize the intersegmental border. In cases where the lesion was deep and ensuring a surgical margin was challenging, preoperative placement of a hook wire was performed in addition to the ICG method. For patients with iodine or ICG allergies, the intersegmental plane was identified using an inflation/deflation line. The intersegmental line was then dissected using a SureForm Stapler via one or two robotic ports. During dissection of the pulmonary hilum, the threads stapled with the vessels and bronchi were pulled to properly separate the pulmonary hilar structures from the remaining lung parenchyma and outline the correct line of dissection (Fig. 2).

### CVATS segmentectomy

CVATS segmentectomies were performed using 3 or 4 ports. The intersegmental border was identified using a fluorescence imaging camera (1688AIM Camera System, Stryker, Kalamazoo, MI, USA) following the same procedure used for RATS. The intersegmental plane was divided using hand-held staplers: Echelon Flex^™^ staplers (Ethicon, Johnson & Johnson, New Brunswick, NJ, USA) or the Endo GIA^™^ stapler (Covidien, Mansfield, MA, USA). The choice between the RATS and CVATS approaches is basically a random assignment, but it is determined by the initial consulting physician and the availability of robotic surgery slots. The indications for both the approaches were identical.

### Statistical analysis

All statistical analyses were performed using the John Macintosh Project (JMP) Pro 16 software program (SAS Institute, Cary, NC, USA). Categorical variables were presented as frequencies and percentages. Continuous variables were expressed as the mean ± standard deviation if normally distributed and median values (range) if not normally distributed. For categorical variables, comparisons between groups were performed using the *χ*2 test or Fisher’s exact test. Continuous variables were compared using the unpaired *t *test or the Mann–Whitney *U* test. The overall survival (OS) and relapse-free survival (RFS) rates were analyzed using the Kaplan–Meier method, and differences between groups were calculated using the log-rank test. Differences were considered statistically significant at *P* < 0.05.

## Results

### The characteristics and surgical outcomes of patients who underwent RATS portal segmentectomy

This study included 50 men and 42 women with a median age of 71 years (range, 37–88 years) who underwent RATS segmentectomy. The patient characteristics and perioperative outcomes are shown in Table 1. Among the patients, 79 were diagnosed with primary lung cancer and 13 were diagnosed with metastatic lung cancer. The surgical procedures included 44 simple segmentectomies and 48 complex segmentectomies. The types of segmentectomy are summarized in Table 2. ICG was used to identify the intersegmental line in 86 patients (93.5%), and preoperative hook-wire placement in addition to ICG was performed in 15 patients. Allergies prevented the use of ICG in five patients (5.5%), and the intersegmental border was identified using an inflation/deflation line. The median operative time was 162 min (range 101–304), and the median console time was 97 min (range 50–221). The mean intraoperative bleeding was 13.9 ml (range 10–100). No intraoperative complications occurred, while postoperative complications were observed in four patients (4.3%), including pneumonia in two patients, arrhythmia in one patient, and prolonged air leak in one patient. The median surgical margin was 20 mm (range 5–60), and the median maximum incision size was 2.5 cm (range 2.0–3.0).

**Table 1:** The characteristics and surgical outcomes of patients who underwent RATS and CVATS segmentectomy

Variables	RATS	CVATS	*P* value
	*n* = 92	*n* = 82	
Patient characteristics			
Age (years old)	71 (37–88)	72 (42–94)	0.969
Gender (male/female)	50/42	47/35	0.694
BMI	23.2 ± 3.5	22.2 ± 3.4	0.076
Diagnosis			
Primary lung cancer	79 (85.9%)	67 (80.5%)	0.342
CTR	0.60 (0–1)	0.64 (0–1)	0.533
CT findings			
Pure solid	26	26	0.508
Mixed GGN	47	34	
Pure GGN	6	7	
c-stage			
0	6	7	0.482
IA	72	56	
IB	1	3	
IIA	0	1	
p-stage			
0	24	13	0.554
IA	53	51	
IB	2	2	
III	0	1	
Metastatic lung cancer	13 (14.1%)	16 (19.5%)	
Tumor size (cm)	1.3 (0.3–4.5)	1.4 (0.5–6)	0.563
%VC	94.9 (58.5–144.2)	90.0 (58.5–117.5)	0.051
%FEV1.0	94.6 (35.3–125.7)	90.6 (49.0–137.4)	0.906
Comorbid lung disease			
COPD	2 (2.2%)	7 (8.5%)	0.238
Interstitial pneumonia	2 (2.2%)	3 (3.7%)	
Asthma	1 (1.1%)	3 (3.7%)	
Permanent tracheal stoma	1 (1.1%)	1 (1.2%)	
Procedure			
Simple segmentectomy	44 (47.8%)	44 (53.7%)	0.442
Complex segmentectomy	48 (52.2%)	38 (46.3%)	
Identification of the intersegmental line			
ICG + hookwire	15 (16.5%)	8 (9.8%)	0.092
ICG	71 (78.0%)	63 (76.8%)	
Inflation/deflation	4 (4.4%)	11 (13.4%)	
Hookwire + inflation/deflation	1 (1.1%)	0	
LN dissection			
≦ND1	78	63	0.182
≧ND2	14	19	
Perioperative outcomes			
Operative time (min)	162 (101–304)	192 (91–313)	0.003
Console time (min)	97 (50–221)	–	
Intraoperative bleeding (mL)	13.9 ± 13.9	24.6 ± 2.7	0.002
Intraoperative complications	0	0	–
Postoperative complications	4 (4.3%)	16 (19.5%)	0.002
Prolonged air leak	1	9	
Arrhythmia	1	3	
Pneumonia	2		
Chylothorax		2	
Hoarseness		1	
Wound infection		1	
Number of staplers	9 (6–15)	7 (4–12)	< 0.001
Surgical margin (mm)	20 (5–60)	20 (5–55)	0.799
Maximum incision size (cm)	2.5 (2.0–3.0)	3.0 (2.0–3.5)	< 0.001

**Table 2:** Features of segmentectomies

	RATS				CVATS			
	Right		Left		Right		Left	
Simple	S6	10	S1 + 2 + 3	15	S6	6	S1 + 2 + 3	16
	S7 + 8 + 9 + 10	6	S4 + 5	5	S7 + 8 + 9 + 10	3	S4 + 5	8
			S6	4			S6	5
			S8 + 9 + 10	4			S8 + 9 + 10	5
Complex	S1	8	S1 + 2	10	S1	3	S1 + 2	13
	S2	5	S3	3	S2	1	S3	4
	S3	3	S3 + 4 + 5	2	S3	2		
	S1 + 2	1	S4	1				
	S3 + 4 + 5	1						
	S8	1	S8	5	S8	2	S8	2
	S10	1	S8 + 9	1	S9	1	S8 + 9	1
	S6 + 10	1	S10	2	S10	1	S9 + 10	2
	S9 + 10	2			S8 + 9	1	S10	1
	S8 + 9 + 10	1			S9 + 10	4	LS*	1

### Comparison of the surgical outcomes between RATS and CVATS segmentectomies

The patient characteristics for CVATS are also shown in Table 1, and did not exhibit any significant differences compared with RATS. The operative time for RATS was significantly shorter than that for CVATS (162 min and 192 min, respectively; *P* = 0.003). Intraoperative bleeding during RATS was significantly less than that during CVATS (13.9 and 24.6 mL, respectively, *P* = 0.002). The incidence of postoperative complications after RATS was significantly lower than that after CVATS (4.3% and 19.5%, respectively; *P* = 0.002). The maximum incision size for RATS was significantly smaller than that for CVATS (2.5 and 3.0 cm, respectively; *P* < 0.001). In contrast, RATS required a higher number of staplers than CVATS (9 and 7, respectively, *P* < 0.001). There were no significant differences in the surgical margins between the RATS and CVATS groups (*P* = 0.799). The median follow-up period in patients with NSCLC who underwent RATS and CVATS was 17.6 months (range 1.5–42.4 months) and 24.3 months (range 1.4–44.1 months), respectively. A comparison of the characteristics of the patients with NSCLC who underwent RATS and CVATS segmentectomies is shown in Table 3. The 2-year OS rate of RATS was 97.1%, which was not significantly different from that of CVATS (89.4%, *P* = 0.161) (Supplemental Fig. 1a). The 2-year RFS rate of RATS was 98.7%, which was not significantly different from that of CVATS (83.6%, *P* = 0.093) (Fig. 1b). There was no recurrence in the patients who underwent RATS segmentectomy. Among the patients who underwent CVATS, there were two cases of multiple pulmonary metastases and one case of pleural dissemination. However, no local recurrence was observed in either RATS or CVATS.

**Table 3:** Comparison of the characteristics between patients with NSCLC who underwent RATS and CVATS segmentectomies

Variables	RATS	CVATS	*P* value
	*n* = 78	*n* = 67	
Age (years old)	72 (37–88)	72 (42–94)	0.902
Gender (male/female)	42/36	39/28	0.600
BMI	22.9 ± 3.5	22.3 ± 3.5	0.174
CTR	0.59 (0–1)	0.64 (0–1)	0.475
Pathology			
Adenocarcinoma	77	59	0.005
Squamous cell carcinoma		8	
Large cell carcinoma	1		
c-stage			
0	6	7	0.490
IA	71	56	
IB	1	3	
IIA		1	
p-stage			
0	24	13	0.554
IA	52	51	
IB	2	2	
III	0	1	
Tumor size (cm)	1.3 (0.5–4.5)	1.5 (0.5–3.2)	0.321
Procedure			
Simple segmentectomy	40 (51.3%)	37 (55.2%)	0.635
Complex segmentectomy	38 (48.7%)	30 (44.8%)	
LN dissection			
≦ND1	66	49	0.089
≧ND2	12	18	
Perioperative outcomes			
Operative time (min)	163.5 (101–304)	191 (91–313)	0.005
Console time (min)	98.5 (50–205)	-	
Intraoperative bleeding (mL)	14.0 ± 14.5	25.2 ± 34.4	0.004
Intraoperative complications	0	0	–
Postoperative complications	4 (5.1%)	13 (19.4%)	0.008
Surgical margin (cm)	22 (5–60)	22 (10–55)	0.550

### Comparison of the surgical outcomes between RATS simple and complex segmentectomies

Table 4 presents a comparison of the patient characteristics and perioperative outcomes between the 44 patients who underwent RATS simple segmentectomies and 48 patients who underwent complex segmentectomies. The patient characteristics did not differ between the simple and complex RATS segmentectomies. The operative time, console time, intraoperative bleeding, intraoperative complications, postoperative complications, number of staplers, and surgical margins were also comparable between simple and complex RATS segmentectomies. However, the maximum incision size for RATS complex segmentectomies was significantly smaller than that for RATS simple segmentectomies (2.0 and 2.5 cm, respectively, *P* = 0.046).

**Table 4:** Comparison of the characteristics between RATS simple and complex segmentectomies

Variables	Simple	Complex	*P* value
	*n* = 44	*n* = 48	
Age (years old)	73.5 (61–88)	70 (37–86)	0.0314
Gender (male)	24 (54.6%)	26 (54.2%)	0.9709
BMI	23.2 ± 3.7	23.1 ± 3.3	0.7987
Diagnosis			
Primary lung cancer	40 (90.9%)	39 (81.3%)	0.1840
Metastatic lung cancer	4 (9.1%)	9 (18.8%)	
Tumor size (cm)	1.2 (0.3–3.8)	1.4 (0.7–4.5)	0.2338
Identification of the intersegmental line			
ICG + VATS marker	7 (15.9%)	8 (17.0%)	0.5526
ICG	34 (77.3%)	37 (78.7%)	
VATS marker	0	1 (2.1%)	
Inflation/deflation	3 (6.8%)	1 (2.1%)	
Perioperative outcomes			
Operative time (min)	161 (101–304)	169 (105–296)	0.8738
Console time (min)	97 (50–217)	97 (50–221)	0.8845
Intraoperative bleeding (mL)	10 (10–100)	10 (10–100)	0.1602
Intraoperative complications	0	1 (2.1%)	0.3357
Postoperative complications	3 (6.8%)	1 (2.1%)	
Pneumonia	1	1	0.2660
Arrhythmia	1		
Prolonged air leak	1		
Number of staplers	9 (6–13)	9 (6–15)	0.1640
Surgical margin (mm)	20 (5–45)	25 (5–60)	0.2488
Maximum incision size (cm)	2.5 (2.0–3.0)	2.0 (2.0–3.0)	0.0460

## Discussion

The utility of RATS in pulmonary segmentectomies has been extensively documented [7–11], and several studies have compared RATS segmentectomy with VATS (Table 5). However, to the best of our knowledge, no previous study has specifically examined the advantages of using a robotic stapler during RATS segmentectomy. Our study demonstrated the safety and efficacy of dividing the intersegmental plane using a robotic stapler in RATS segmentectomy.

**Table 5:** Published studies comparing RATS segmentectomy to VATS in over 30 cases

Author	Year	Arms	Number of patients	Da Vinci	Operative time	*p*	Conversion	*p*	Prolonged air leak	*p*	Stapler
Demir	2014	RATS	34	S/Si	76	0.018	2.9%	0.66	NA	–	Hand-held
		VATS	65		65		4.6%		NA		Hand-held
Xie	2019	RATS	42	NA	126.2	0.118	NA	–	4.6%	0.569	Hand-held
		VATS	46		124.1		NA		6.5%		Hand-held
Zhang	2020	RATS	257	S	147.9	0.773	0.4%	0.624	4.3%	0.624	Hand-held
		VATS	257		149.2		1.2%		1.9%		Hand-held
Yang	2023	RATS	132	Si/Xi	111.4	0.014	0%	NA	3.0%	NA	NA
		VATS	167		133.6		1.2%		1.8%		Hand-held
Haruki	2023	RATS	63	S/Si/ X/XI	154	< 0.001	NA		5%	0.649	Hand-held Robotic
		VATS/ Open	63		210		NA		3%		Hand-held
Present study	2023	RATS	92	Xi	162	0.0030	0%	–	1.1%	0.0014	Robotic
		CVATS	82		192		0%		11.0%		Hand-held
NA, not applicable											

In a randomized controlled trial, it was found that division of the intersegmental plane using a stapler reduced postoperative complications compared to division using electrocautery during pulmonary segmentectomy [12]. Moreover, there was no difference in the respiratory function between the electrocautery and stapler groups. Several retrospective studies have also reported a higher incidence of prolonged air leaks in the electrocautery-alone group than in the stapler group, and the use of a stapler did not result in a significant loss of preserved lung volume function compared with electrocautery [13, 14]. Based on these findings, it is likely that the division of the intersegmental plane using a stapler will become the standard practice. Furthermore, as segmentectomy becomes more prevalent, the number of cases that require ipsilateral reoperation after segmentectomy is expected to increase. However, such procedures are often challenging to perform [15]. Specifically, if the segmental plane is dissected by electrocautery and extensively covered with fibrin glue and PGA sheets, adhesions become extensive and severe, thus further complicating reoperation [16, 17].

In the present study, the operative time and blood loss during RATS segmentectomy were significantly lower than those during CVATS. Although most previous studies have reported that the operative time for RATS segmentectomy was either longer or comparable to that for VATS segmentectomy [7, 8, 18], these studies seem to have used hand-held staplers without employing a robotic stapler. The use of a robotic stapler may have contributed to the shorter operative time in RATS. However, many previous reports did not specify whether CVATS or hybrid VATS was performed, whereas this study exclusively compared CVATS with RATS. This could be another contributing factor because the limitation in the stapler insertion direction makes it technically more challenging to use staplers than hybrid VATS.

The postoperative complications following RATS were significantly lower than those following CVATS. In particular, the occurrence of prolonged air leaks was noticeably reduced in RATS compared to CVATS. This can be attributed to several factors, including the ability of RATS to provide precise exposure of hilar structures through enhanced 3D and magnified vision as well as its capacity for accurate dissection along intersegmental lines using the SureForm stapler. One advantage of the SureForm stapler is its increased flexibility during bending. While hand-held staplers allow only 45° to the left and right, SureForm permits bending of up to 60°in all directions, including both the lateral and vertical orientations. This versatility in stapler bending is particularly beneficial because it enables stapling from only one or two ports, even in complex segmentectomies. CO_2_ insufflation is important for the comfortable use of SureForm because it expands the thoracic cavity and provides a wide working space to effectively utilize SureForm. However, it should be noted that the use of SureForm may pose some technical challenges in patients with a short stature. The stapling site of SureForm must be positioned within the port in the thoracic cavity; however, in individuals with a short stature, the stapling site may sometimes be too close to the port, thus making it difficult to achieve the desired bending of the SureForm stapler.

Several key points were involved in dissecting the intersegmental plane along the correct line. First, it is important to clearly delineate the intersegmental line. Near-infrared fluorescence mapping using ICG has recently been reported as a valuable technique for identifying the intersegmental plane [19, 20]. Near-infrared fluorescence mapping using ICG can be easily performed using a Firefly Fluorescence Imaging camera, which is beneficial during RATS segmentectomies. Additionally, when the intersegmental plane was close to the tumor, we also utilized hook-wire localization. Although the hook itself cannot be visualized, the location of the insertion site of the wire can assist in identifying intersegmental lines, such as when it is difficult to identify intersegmental lines using ICG. In addition, if the hook is confirmed to be included in the resected specimen, it is a marker for tumor resection. Second, to facilitate easy division of the intersegmental plane at the pulmonary hilum using a stapler, it is necessary to adequately dissect the blood vessels and bronchi at the hilum, pushing them as far peripherally as possible using a bipolar device. Utilizing the advantages of the robot, such as 3D visualization and magnification, can help prevent air leakage after the procedure. Third, visualizing the line to be dissected with the robotic stapler involves sufficient traction on the resected vessels and bronchi in the pulmonary hilum. The silk threads used for taping blood vessels and bronchi were intentionally stapled together with these structures using a stapler. These threads were pulled to widen the view of the pulmonary hilum and outline the correct dissection line, as shown in Fig. 1. The retraction arm is utilized to pull these sutures, and it is a useful technique that takes advantage of the features of the robot. The retraction arm could be easily moved in various directions and it did not interfere with the field of view. Instead of pulling the thread, forceps can be used to directly grasp the vessels and bronchi; however, the forceps are in close proximity to the stapler during stapling and interfere with the stapler. Failure to perform these procedures can result in the presence of residual pulmonary hilar structures that should be resected or a narrowing of remaining blood vessels, thus leading to congestion and other complications.

We also compared complex segmentectomies with simple segmentectomies in the RATS cases. Complex segmentectomy is generally considered more technically challenging than simple segmentectomy and presents difficulties in achieving a sufficient surgical margin. However, the operative time, console time, surgical margin, and incidence of perioperative complications were comparable between RATS complex and simple segmentectomy. These findings suggest that RATS complex segmentectomy can be safely performed using a robotic stapler. Complex segmentectomy requires the peripheral dissection of the vascular structures and bronchi within the lung parenchyma, and there is a high risk of structural misidentification. Therefore, whether the vessels and bronchi should be dissected should be carefully determined prior to dissection. To address this, the surrounding vessels and bronchi are exposed as much as possible before dissection, and the vessels and bronchi are dissected after a good understanding of the overall structure. 3D visualization and magnification, which are the advantages of the robot, can help with these procedures.

One of the advantages of RATS portal segmentectomy is its cosmetic aspect. While CVATS and RATS with an assist window usually require a 3 cm or more incision, RATS portal segmentectomy utilizes the minimum necessary incision for lung extraction, resulting in a significantly smaller incision compared to CVATS. Furthermore, in RATS portal complex segmentectomy, where the resected lung is smaller than in RATS portal simple segmentectomy, the maximum incision size is also significantly smaller.

Cost is often cited as a recurring issue in the RATS. In the present study, RATS segmentectomy using a robotic stapler required significantly more staplers than CVATS segmentectomy, thus suggesting that RATS segmentectomy using a robotic stapler costs more than CVATS segmentectomy. However, a reduction in the operative time and postoperative complications may also lead to cost savings. In fact, recent studies comparing the cost of RATS and VATS segmentectomies reported that the costs were comparable between RATS and VATS segmentectomies [8, 21]. Zervos et al. compared the use of robotic and handheld staplers during RATS lobectomy and reported that the robotic stapler significantly reduced perioperative complications, and the total index hospitalization costs were comparable [22]. The reason why a large number of staplers are required in RATS is likely because we use the 45 mm stapler to dissect the intersegmental plane. The SureForm stapler is a disposable product with a maximum capacity of 12 firings and requires the choice of a 45 or 60 mm option. To reduce the number of staplers, both 45 mm and 60 mm SureForm staplers could also be used, but this would incur additional costs for the main unit and may not yield significant cost-saving benefits. Moreover, it is challenging to use the 60 mm SureForm for all vascular and lung dissections, so we exclusively use the 45 mm SureForm stapler. Recently, we have been conserving staplers by ligating the vessels instead of using them because segmentectomies often involve the dissection of small vessels.

### Limitations

This study is associated with several limitations. First, this was a retrospective study, and a prospective randomized study is required in the future. Second, we were not able to compare the effectiveness of the robotic and hand-held staplers in RATS. Third, 21 cases of CVATS were performed by trainees, whereas RATS was performed by only attending surgeons. Therefore, we reanalyzed the surgical outcomes, excluding cases performed by trainee surgeons, as shown in Supplemental Table 2. However, the perioperative results were similar to those of the main analysis even when trainee surgeons were excluded. Finally, the objective of this study was to obtain the short-term perioperative outcomes; a longer follow-up period is necessary to clarify the efficacy of RATS segmentectomy using a robotic stapler.

## Conclusion

The division of the intersegmental plane using a robotic stapler in RATS portal segmentectomy was found to be safe and effective, including complex segmentectomy. Although RATS required a significantly higher number of staplers than CVATS, RATS yielded superior intraoperative and postoperative short-term outcomes when compared to CVATS.

## Supplementary Information

Below is the link to the electronic supplementary material.

Supplementary file1 (DOCX 33 KB)

Supplementary file2 (JPG 70 KB)
