# Supplementary material for: Effective division of the intersegmental plane using a robotic stapler in robotic pulmonary segmentectomy
Source: Surg Today. 2024 Apr 18;54(11):1319–28. doi: 10.1007/s00595-024-02840-y (PMC11499527; doi:10.1007/s00595-024-02840-y)
Supplement: Supplementary file 1 — Supplementary file1 (DOCX 33 KB) [file 595_2024_2840_MOESM1_ESM.docx]

| Supplemental TABLE 1: The characteristics and surgical outcomes of patients who underwent RATS and CVATS segmentectomy by attending surgeons | | | |
| --- | --- | --- | --- |
| Variables | RATS | CVATS | *P*-value |
|  | n = 92 | n =61 |  |
| Patient characteristics |  |  |  |
| Age (years old) | 71 (37-88) | 72 (42-94) | 0.816 |
| Gender (male/female) | 50/42 | 29/32 | 0.409 |
| BMI | 23.2 ± 3.5 | 21.9 ± 3.2 | 0.024 |
| Diagnosis |  |  | 0.473 |
| Primary lung cancer | 79 (85.9%) | 49 (80.3%) |  |
| c-Stage |  |  | 0.382 |
| 0 | 6 | 5 |  |
| IA | 72 | 41 |  |
| IB | 1 | 2 |  |
| IIA | 0 | 1 |  |
| Metastatic lung cancer | 13 (14.1%) | 12 (19.7%) |  |
| Tumor size (cm) | 1.3 (0.3-4.5) | 1.4 (0.5-6) | 0.441 |
| Procedure |  |  | 0.338 |
| Simple segmentectomy | 44 (47.8%) | 34 (55.7%) |  |
| Complex segmentectomy | 48 (52.2%) | 27 (44.3%) |  |
| Perioperative outcomes |  |  |  |
| Operative time (min) | 162 (101-304) | 190 (91-299) | 0.014 |
| Console time (min) | 97 (50-221) | - |  |
| Intraoperative bleeding (mL) | 13.9±13.9 | 23.8±3.1 | 0.014 |
| Intraoperative complications | 0 | 0 | - |
| Postoperative complications | 4 (4.3%) | 12 (19.7%) | 0.002 |
| Prolonged air leak | 1 | 7 |  |
| Arrhythmia | 1 | 3 |  |
| Pneumonia | 2 |  |  |
| Chylothorax |  | 2 |  |
| Hoarseness |  | 1 |  |
| Number of staplers | 9 (6-15) | 6.5 (4-12) | <0.001 |
| Surgical margin (mm) | 20 (5-60) | 20 (6-55) | 0.980 |
| Maximum incision size (cm) | 2.5 (2.0-3.0) | 3.0 (2.0-3.5) | <0.001 |
|  |  |  |  |
|  |  |  |  |
