# Supplementary figures and images for: Effective division of the intersegmental plane using a robotic stapler in robotic pulmonary segmentectomy
Source: Surg Today. 2024 Apr 18;54(11):1319–28. doi: 10.1007/s00595-024-02840-y (PMC11499527; doi:10.1007/s00595-024-02840-y)

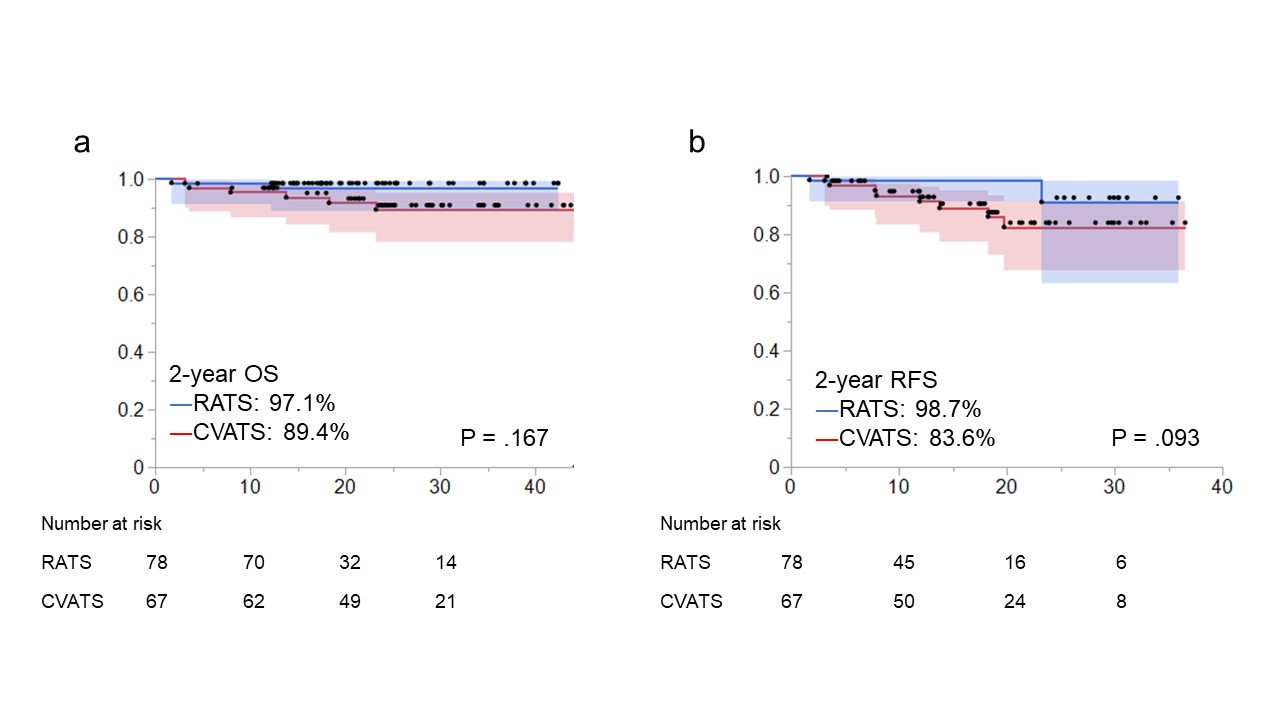

Supplement: Supplementary file 2 — Supplementary file2 (JPG 70 KB) [file 595_2024_2840_MOESM2_ESM.jpg]
